# Circulating Illness and Changes in Thermometer Use Behavior: Series of Cross-sectional Analyses

**DOI:** 10.2196/37509

**Published:** 2022-09-08

**Authors:** Jack Seifarth, Megan Pinaire, John Zicker, Inder Singh, Danielle Bloch

**Affiliations:** 1 Department of Epidemiology Mailman School of Public Health Columbia University New York, NY United States; 2 Kinsa Inc San Francisco, CA United States; 3 Department of Biostatistics Yale School of Public Health New Haven, CT United States

**Keywords:** thermometer, health behavior, influenza, COVID-19, fever, surveillance, perceived risk, percent positivity, smart technology, smart thermometer, population demography, older adult, elderly population, health monitoring

## Abstract

**Background:**

Temperature-taking behaviors vary with levels of circulating infectious illness; however, little is known about how these behaviors differ by demographic characteristics. Populations with higher perceived risks of illness are more likely to adopt protective health behaviors.

**Objective:**

We investigated differences in temperature-taking frequency and the proportion of readings that were feverish among demographic groups (age, gender, urban/rural status) over influenza offseason; influenza season; and waves 1, 2, and 3 of the COVID-19 pandemic.

**Methods:**

Using data from smart thermometers collected from May 1, 2019, to February 28, 2021, across the United States, we calculated the frequency of temperature-taking and the proportion of temperature readings that were feverish. Mixed-effects negative binomial and mixed-effects logistic regression analyses were performed to identify demographic characteristics associated with temperature-taking frequency and the proportion of feverish readings, respectively. Separate models were fit over five study periods: influenza offseason (n=122,480), influenza season (n=174,191), wave 1 of COVID-19 (n=350,385), wave 2 (n=366,489), and wave 3 (n=391,578).

**Results:**

Both temperature-taking frequency and the proportion of feverish readings differed by study period (ANOVA *P*<.001) and were the highest during influenza season. During all periods, children aged 2-5 years and 6-11 years had significantly higher frequencies of temperature-taking than users aged 19-30 years, and children had the highest proportion of feverish readings of all age groups, after adjusting for covariates. During wave 1 of COVID-19, users over the age of 60 years had 1.79 times (95% CI 1.76-1.83) the rate of temperature-taking as users aged 19-30 years and 74% lower odds (95% CI 72%-75%) of a reading being feverish. Across all periods, men had significantly lower temperature-taking frequency and significantly higher odds of having a feverish reading compared to women. Users living in urban areas had significantly higher frequencies of temperature-taking than rural users during all periods, except wave 2 of COVID-19, and urban users had higher odds of a reading being feverish in all study periods except wave 1 of COVID-19.

**Conclusions:**

Temperature-taking behavior and the proportion of readings that were feverish are associated with both population disease levels and individual demographic characteristics. Differences in the health behavior of temperature-taking may reflect changes in both perceived and actual illness risk. Specifically, older adults may have experienced an increase in perceived risk during the first three waves of COVID-19, leading to increased rates of temperature monitoring, even when their odds of fever were lower than those of younger adults. Men’s perceived risk of circulating infectious illnesses such as influenza and COVID-19 may be lower than that of women, since men took their temperature less frequently and each temperature had a higher odds of being feverish across all study periods. Infectious disease surveillance should recognize and incorporate how behavior impacts illness monitoring and testing.

## Introduction

At-home health monitoring behaviors have the potential to greatly impact health outcomes. However, health behaviors differ by demographic and social determinants, including poverty, gender, and neighborhood social and physical characteristics [1]. For example, women and older individuals are more likely to report practicing protective health behaviors [2] such as taking COVID-19 precautions [3]. Individuals also alter behaviors in response to circulating illness levels and associated health recommendations or policies; this was observed with both increases in mask wearing and social distancing after the H1N1 influenza outbreak [4], and increases in handwashing and social distancing during the COVID-19 pandemic [5].

The Health Action Process Approach (HAPA) framework for health behavior states that perceived risk, particularly of severe health outcomes, can motivate health behavior change [6,7]. Additionally, changes to perceived risk based on underlying conditions, attention to media coverage, or knowledge of disease can impact health behaviors [8,9]. A study of behavior among individuals in the United States during spring of 2020 guided by HAPA found that risk perception and self-efficacy were both predictors of social distancing [10]. A systematic review of nonpharmaceutical interventions prior to the COVID-19 pandemic found that individuals adopt behaviors partially based on their perceived vulnerability of respiratory illness [11].

Most respiratory illness-related health behavior studies are cross-sectional surveys relying on self-reported behavior during a pandemic or influenza season [12-14]. Temperature-taking using a smart thermometer is a timely and sensitive surveillance measure that circumvents the issue of self-reporting. Smart thermometers record body temperatures and aggregate the anonymized, deidentified readings along with basic demographic information [15]. Based on a user’s temperature, symptoms, and age, they receive illness guidance through an associated app. Aggregated user temperature data are closely correlated with traditional influenza surveillance methods [16-18]. Unlike COVID-19 prevention behaviors such as mask wearing and social distancing, temperature-taking is not typically performed in public settings. Therefore, temperature-taking may be less influenced by social pressures and could therefore better approximate individual perceptions of risk. Previous studies using smart thermometers have shown that the number of fevers and the total number of thermometer readings correlate with influenza-like illness at the national and regional levels during both influenza season [16,17] and offseason [16], and with influenza test positivity at the regional level [17]. Kinsa fever data were also found to be correlated with confirmed cases during the first wave of COVID-19 [19].

The demographic and social determinants of the health behavior of temperature-taking have yet to be examined. We predicted that demographic groups with greater perceived risks of influenza or COVID-19 will take their temperature more often. Using data from smart thermometer users collected between May 1, 2019, and February 28, 2021, we aimed to understand how both demographic characteristics and external circulating illness were associated with temperature-taking frequency and the percent of readings with a fever. We use temperature-taking frequency as a proxy for an individual’s perceived risk of illness and percent feverish readings to determine if there was febrile illness in the presence of that concern. We sought to explore how both social determinants of health and external influenza and COVID-19 levels impacted perceived and actual risks of febrile illness.

## Methods

### Data Collection

Kinsa smart thermometers record and store body temperatures using a smartphone app. Most users purchase their thermometer through major retailers. Kinsa thermometers are also distributed free of charge for families in Title 1 elementary schools through a program called FLUency [20]. Title 1 programs provide federal funding to schools with high numbers or percentages of children from low-income families [21]. FLUency school nurses can use the program to communicate with families about current illness in the school or grade. When any user takes a temperature, the reading and timestamp are recorded along with deidentified, user-entered demographic information, including age and gender. Readings are geocoded using GPS coordinates or the IP address of the connected device. Users can assign temperature readings to different profiles within their account, allowing for differentiation among readings from multiple users in the same household.

### Study Population

Individuals who recorded at least one temperature reading with a Kinsa thermometer in the United States from May 1, 2019, to February 28, 2021, were included in this analysis. Study periods were defined based on trends of seasonal influenza and COVID-19: influenza offseason (May 1, 2019, to October 31, 2019), influenza season (November 1, 2019, to February 2, 2020), wave 1 of COVID-19 (February 3, 2020, to May 31, 2020), wave 2 of COVID-19 (June 1, 2020, to October 31, 2020), and wave 3 of COVID-19 (November 1, 2020, to February 28, 2021). The 2019-2020 influenza season was considered moderately severe and was dominated by A(H1N1)pdm09 viruses [22]. The influenza season ended in early 2020, likely due to COVID-19 lockdowns and precautions [22].

Users were only included in a study period if they recorded at least one temperature reading within that period. Therefore, the same user would not be included in all study periods if they did not record a reading in each separate period.

We performed our analyses on users who had no missing demographic or geographic information. Standardized mean differences showed that the full population had a similar number of readings as the complete case population.

### Measures

The two outcomes assessed were: (1) the number of readings per user and (2) the proportion of readings with a fever. The number of readings provides a measure of how often a user is potentially concerned about a possible fever and the proportion of readings with a fever provides a measure of how many readings were taken because of a true fever. To define the number of readings per user, we counted each reading a user took during a given period and then adjusted for varying amounts of follow-up time. The number of days a user was active during a study period was calculated from device activation through to the end of that period. If the activation date was before the start of the study period, the user was considered to be active for the entire period. Because users could take multiple readings on the same day, a sensitivity analysis was performed that examined the number of distinct days with at least one temperature reading to circumvent intraday variability in reading behavior. The second outcome, proportion of readings with a fever, was defined as the number of readings >37.8°C divided by the total number of readings for a user during a period.

Age and gender were self-reported and defined at the first reading during the period. Age was categorized into 0-1 years, 2-5 years, 6-11 years, 12-18 years, 19-30 years, 31-60 years, and 61+ years. We assumed that an adult in the household was driving temperature-taking among individuals 18 years and under. Any user associated with a device that was distributed through the school program was categorized as a FLUency user.

Household composition was derived from the ages of registered users associated with one thermometer: if all users were <18 years old, the household was considered “child only”; if all users were aged 18 years and older, the household was considered “adult only”; and if there were users both under 18 years and 18 years and older, the household was categorized as “multigeneration.” “Child-only” households reflect devices where a parent/guardian has not created a profile for themself but has created profiles for their children.

Neighborhood poverty, US region, and urban/rural designation were determined based on the location with a majority of a user’s readings. Census tract–level poverty was obtained from the 2015-2019 American Community Survey and defined as the percent of residents living below 100% of the federal poverty level [23]. Poverty was then classified into four categories: 0 to <10%, 10% to <20%, 20% to <30%, and ≥30%. Region was defined using the 10 regions created by the Centers for Disease Control and Prevention National Center for Chronic Disease Prevention and Health Promotion [24]. A user was categorized as living in an urban area if 50% or more of the land area of their census tract (based on the 2010 US Census) was classified as urban [25].

### Statistical Analysis

We assessed unadjusted differences in both temperature-taking frequency and the proportion of readings with a fever across periods using ANOVA. Differences in the frequency of the categorical variables across periods were assessed by *χ*^2^ tests.

We used mixed-effects negative binomial models to examine the relationships between explanatory variables and the overdispersed outcome of frequency of readings per month. A separate model was fit for each of the five study periods. Since users were nested within devices, device was treated as a random effect. All other variables of interest were treated as fixed effects. The outcome of the number of readings was offset by the number of days a user was active during the period to obtain a frequency of readings over time. This offset calculated a more conservative estimate of variance. We checked for collinearity among predictor variables by requiring their generalized variance inflation factors to be less than 5. Age group, gender, urban/rural status, census tract poverty group, household composition, region, and FLUency participation were included in the final model. A predictor was considered significant if the 95% CI of its incidence rate ratio (IRR) did not contain the null value of 1. The same methods were applied to the outcome of the distinct number of days with a reading in the sensitivity analysis.

We used mixed-effects logistic (binomial) regression to examine the adjusted relationships between our explanatory variables and the proportion of readings with a fever. A separate model was fit for each of the five study periods with the outcome of number of fevers and number of nonfevers recorded per user. Device was added as a random intercept with all other predictors treated as fixed effects. A predictor was considered significant if the 95% CI of its odds ratio (OR) did not contain the null value of 1.

Statistical analyses were performed in R 4.1.0 (R Core Team, Vienna, Austria 2021) using the glmmTMB package [26]. Reported regression outputs and ratios are adjusted for all predictors included in the model.

### Ethical Considerations

Upon downloading the app, users were asked to acknowledge and consent to data collection practices outlined in the Kinsa Privacy Policy [15]. Users must expressly consent to sharing geolocation data. All personally identifiable information was collected and maintained in compliance with state and federal confidentiality guidelines. This study was approved by an external institutional review board, Advarra Inc (Pro00065469).

## Results

### Descriptive Statistics

There were 122,480 users with full demographic information in influenza offseason 2019, 174,191 users in influenza season 2019-20, 350,385 users during wave 1 of COVID-19, 366,489 users during wave 2, and 391,578 users during wave 3 (Table 1). The combined study population had a median age of 26 years (IQR 6-43), with 57.3% women, and primarily resided in urban (77.8%) and 0-10% poverty tracts (56.0%); 12.9% of users obtained their thermometer through the FLUency program. The study populations changed significantly over time (Table 1). Notably, the median age of the study population increased from 7 years during the influenza offseason to 30 years in wave 1 of COVID-19.

**Figure 1:**
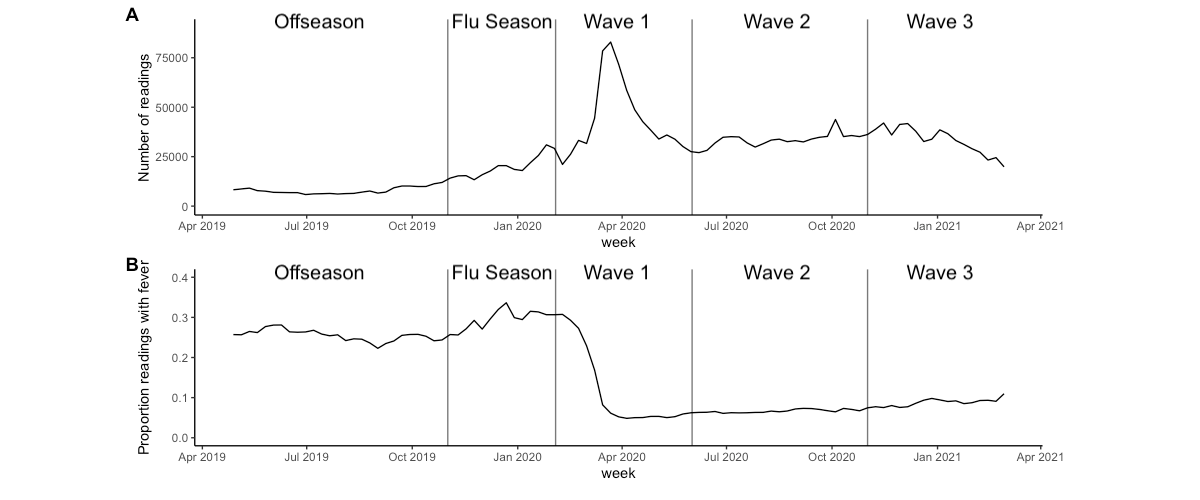
The number of thermometer readings (A) and proportion of readings with a fever (B) aggregated by week, May 2019-February 2021. Study periods are separated by vertical lines (influenza offseason; influenza season; COVID-19 pandemic waves 1, 2, and 3).

Temperature-taking frequency differed significantly by study period (Figure 1; *F*=141.2, *P*<.001). The median frequency of readings was lower during influenza offseason compared to that during influenza season (1.22 vs 2.06 readings per month). During the COVID-19 pandemic, the median reading frequency was the highest during wave 1 (1.79 readings per user per month) and decreased slightly during waves 2 and 3 (1.76 and 1.23 readings per month, respectively).

The proportion of readings with a fever also differed significantly by study period (Figure 1; *P*<.001). The mean percent of readings that were feverish was the highest during the influenza season (21.2%) and was the lowest during wave 2 of COVID-19 (5.6%).

**Table 1:** Demographic characteristics of the study population with temperature readings, stratified by study period, May 2019 to February 2021.

Characteristics		Offseason (May 1, 2019, to October 31, 2019)	Flu season (November 1, 2019, to February 2, 2020)	Wave 1 of COVID-19 (February 3, 2020, to May 31, 2020)	Wave 2 of COVID-19 (June 1, 2020, to October 31, 2020)	Wave 3 of COVID-19 (November 1, 2020, to February 28, 2021)	*P* value	
Users, n		122,480	174,191	350,385	366,489	391,578	N/A^a^	
Devices, n		83,628	113,433	219,056	239,010	255,483	N/A	
Temperature readings per user, median (IQR)		3 (1-8)	3 (1-9)	4 (1-13)	4 (1-12)	3 (1-9)	N/A	
Follow-up time (days), median (IQR)		130 (44-184)	69 (32-94)	79 (63-119)	109 (49-153)	105 (72-120)	N/A	
Temperature readings/month, median (IQR)		1.22 (0.41-3.97)	2.06 (0.72-6.52)	1.79 (0.65-5.75)	1.76 (0.60-5.81)	1.23 (0.51-3.56)	<.001^b^	
Days with a temperature reading, median (IQR)		1 (1-3)	1 (1-3)	2 (1-4)	2 (1-5)	2 (1-4)	N/A	
Days with a temperature reading/month, median (IQR)		0.53 (0.25-1.45)	0.97 (0.46-2.17)	0.81 (0.42-2.10)	0.87 (0.24-1.52)	0.72 (0.34-1.65)	<.001^b^	
Readings with a fever (%), median (IQR)		0 (0-0.30)	0 (0-0.39)	0 (0-0)	0 (0-0)	0 (0-0)	<.001^b^	
Age (years), median (IQR)		7 (2-30)	8 (3-30)	30 (8-48)	28 (9-46)	29 (9-46)	N/A	
**Age group, n (%)**								<.001^c^
	0-1	20,146 (16.4)	17,898 (10.3)	18,914 (5.4)	20,325 (5.5)	18,329 (4.7)		
	2-5	27,520 (22.5)	35,372 (20.3)	38,276 (10.9)	34,784 (9.5)	36,221 (9.3)		
	6-11	22,387 (18.3)	45,924 (26.4)	46,554 (13.3)	47,890 (13.1)	57,647 (14.7)		
	12-18	7127 (5.8)	14,751 (8.5)	23,659 (6.8)	27,857 (7.6)	28,559 (7.3)		
	19-30	13,305 (10.9)	16,312 (9.4)	42,521 (12.1)	58,690 (16.0)	59,279 (15.1)		
	31-60	28,935 (23.6)	40,624 (23.3)	128,326 (36.6)	130,512 (35.6)	139,048 (35.5)		
	61+	3060 (2.5)	3310 (1.9)	52,135 (14.9)	46,431 (12.7)	52,495 (13.4)		
**Gender, n (%)**								<.001^c^
	Women	69,577 (56.8)	100,016 (57.4)	198,125 (56.5)	210,423 (57.4)	226,531 (57.9)		
	Men	52,903 (43.2)	74,175 (42.6)	152,260 (43.5)	156,066 (42.6)	165,047 (42.1)		
**Poverty group^d^, n (%)**								<.001^c^
	0 to <10%	69,099 (56.4)	92,356 (53.0)	207,678 (59.3)	208,457 (56.9)	208,693 (53.3)		
	10 to <20%	36,370 (29.7)	54,457 (31.3)	96,911 (27.7)	100,535 (27.4)	116,984 (29.9)		
	20 to <30%	12,046 (9.8)	19,192 (11.0)	31,512 (9.0)	37,045 (10.1)	42,959 (11.0)		
	≥30%	4965 (4.1)	8186 (4.7)	14,284 (4.1)	20,452 (5.6)	22,942 (5.9)		
**Density^e^, n (%)**								<.001^c^
	Urban tract (%)	96,212 (78.6)	127,192 (73.0)	282,673 (80.7)	291,516 (79.5)	295,074 (75.4)		
	Rural tract (%)	26,268 (21.4)	46,999 (27.0)	67,712 (19.3)	74,973 (20.5)	96,504 (24.6)		
**FLUency group^f^, n (%)**								<.001^c^
	FLUency user	7673 (6.3)	46,809 (26.9)	31,695 (9.0)	33,402 (9.1)	66,930 (17.1)		
	Non-FLUency user	114,807 (93.7)	127,382 (73.1)	318,690 (91.0)	333,087 (90.9)	324,648 (82.9)		
**Household composition^g^, n (%)**								<.001^c^
	Adult-only house	24,170 (19.7)	31,706 (18.2)	167,023 (47.7)	180,067 (49.1)	192,482 (49.2)		
	Child-only house	53,461 (43.6)	78,542 (45.1)	70,015 (20.0)	71,099 (19.4)	77,866 (19.9)		
	Multigenerational house (%)	44,849 (46.6)	63,943 (36.7)	113,347 (32.3)	115,323 (31.5)	121,230 (31.0)		
**Region^h^ (comprising state abbreviations), n (%)**								<.001^c^
	1 (CT, ME, RI, MA, NH, NY, VT)	11,511 (9.4)	14,681 (8.4)	39,386 (11.2)	53,182 (14.5)	44,391 (11.3)		
	2 (DC, MD, WV, DE, NJ, PA, VA)	15,474 (12.6)	21,808 (12.5)	44,449 (12.7)	40,462 (11.0)	63,308 (16.2)		
	3 (GA, FL, NC, SC)	17,207 (14.0)	22,567 (13.0)	40,731 (11.6)	37,444 (10.2)	37,389 (9.5)		
	4 (KY, TN, AL, MS)	5918 (4.8)	10,579 (6.1)	12,699 (3.6)	12,317 (3.4)	17,136 (4.4)		
	5 (IL, WI, IN, MI, MN, OH)	19,509 (15.9)	28,607 (16.4)	57,017 (16.3)	61,453 (16.8)	61,028 (15.6)		
	6 (OK, AR, LA, NM, TX)	17,547 (14.3)	28,915 (16.6)	39,731 (11.3)	38,069 (10.4)	45,840 (11.7)		
	7 (NE, IA, KS, MO)	7053 (5.8)	11,578 (6.6)	16,867 (4.8)	18,975 (5.2)	18,531 (4.7)		
	8 (MT, ND, WY, CO, SD, UT)	3345 (2.7)	4343 (2.5)	12,129 (3.5)	13,998 (3.8)	13,627 (3.5)		
	9 (CA, NV, AZ, HI)	20,990 (17.1)	26,310 (15.1)	70,611 (20.2)	73,715 (20.1)	75,880 (19.4)		
	10 (AK, ID, OR, WA)	3926 (3.2)	4803 (2.8)	16,765 (4.8)	16,874 (4.6)	14,448 (3.7)		

^a^N/A: not applicable.

^b^Differences in continuous variables across study periods assessed via ANOVA.

^c^Differences in frequencies of categorical variables across study periods assessed via *χ*^2^ test.

^d^Percentage of population living below the 100% federal poverty level at the census tract level, from the 2015-2019 American Community Survey.

^e^Categorized as urban if the census tract was part of an urbanized area of 50,000 or more people based on the 2010 US Census.

^f^Received the thermometer through Kinsa’s school distribution and engagement program, FLUency.

^g^Based on ages of profiles associated with the device, with child-only households representing devices where a parent has made profiles for their children but not themself.

^h^Classified using the Centers for Disease Control and Prevention National Center For Chronic Disease Prevention and Health Promotion Regions.

### Temperature-Taking Frequency Regression

During all study periods, the age groups of 0-1, 2-5, and 6-11 years had significantly higher rates of temperature-taking than those of users aged 19-30 years (Table 2). During influenza season, users aged 6-11 years had elevated rates of temperature-taking (IRR 2.25, 95% CI 2.18-2.31) and users aged over 60 years had suppressed rates of temperatures-taking (IRR 0.94, 95% CI 0.89-1.00) compared to users aged 19-30 years. During wave 1 of the COVID-19 pandemic, these age patterns reversed, with users aged over 60 years taking temperatures at a significantly increased rate (IRR 1.79, 95% CI 1.76-1.83) compared to those of young adults. During COVID-19 waves 2 and 3, users aged over 60 years continued to have significantly higher frequencies of temperature-taking (Table 2).

Men had a significantly lower rate of temperature-taking compared to women during all periods (Table 2), and the difference was the largest during influenza offseason (IRR 0.91, 95% CI 0.90-0.93). Users living in urban census tracts had an increased rate of temperature-taking compared to that of rural users during all periods except wave 2 of COVID-19, when urban users had 0.95 (95% CI 0.93-0.96) times the rate of temperature-taking compared to rural users. Similar trends for age, gender, and population density were observed in the sensitivity analysis using the outcome of distinct days with at least one reading (Multimedia Appendix 1).

**Table 2:** Characteristics associated with temperature-taking frequency from multivariable mixed-effects negative binomial regressions, May 2019-February 2021.

Variable			Incidence rate ratio (95% CI)^a^								
			Offseason 2019 (May 1, 2019, to October 31, 2019)		Flu season (November 1, 2019, to February 2, 2020)		Wave 1 of COVID-19 (February 3, 2020, to May 31, 2020)		Wave 2 of COVID-19 (June 1, 2020, to October 31, 2020)		Wave 3 of COVID-19 (November 1, 2020, to February 28, 2021)
**Age (ref: 19-30 years)**											
	0-1 years	2.41 (2.33-2.50)		1.65 (1.59-1.70)		1.78 (1.73-1.83)		1.34 (1.30-1.37)		1.36 (1.32-1.39)	
	2-5 years	2.37 (2.29-2.45)		2.23 (2.16-2.29)		1.71 (1.67-1.74)		1.26 (1.23-1.29)		1.29 (1.26-1.31)	
	6-11 years	1.90 (1.84-1.97)		2.25 (2.18-2.31)		1.40 (1.37-1.43)		1.11 (1.09-1.14)		1.08 (1.06-1.10)	
	12-18 years	1.40 (1.34-1.47)		1.67 (1.61-1.72)		1.03 (1.01-1.06)		0.94 (0.92-0.96)		0.92 (0.90-0.94)	
	31-60 years	0.92 (0.90-0.95)		0.96 (0.93-0.99)		1.32 (1.30-1.34)		1.02 (1.01-1.04)		1.08 (1.07-1.10)	
	>60 years	1.05 (0.98-1.11)		0.94 (0.89-1.00)		1.79 (1.76-1.83)		1.28 (1.25-1.31)		1.28 (1.26-1.31)	
Gender, men (ref: women)			0.91 (0.90-0.93)		0.94 (0.92-0.95)		0.93 (0.92-0.94)		0.96 (0.95-0.97)		0.95 (0.94-0.96)
Density, urban (ref: rural)^b^			1.06 (1.03-1.09)		1.12 (1.09-1.14)		1.08 (1.06-1.10)		0.95 (0.93-0.96)		1.03 (1.02-1.04)
**Poverty (ref: 0 to <10%)^c^**											
	10% to <20%	0.98 (0.95-1.00)		0.98 (0.96-1.00)		1.03 (1.01-1.04)		1.02 (1.00-1.03)		1.00 (0.99-1.02)	
	20% to <30%	0.97 (0.94-1.01)		0.97 (0.94-1.00)		1.07 (1.05-1.09)		0.99 (0.97-1.01)		0.95 (0.93-0.97)	
	≥30%	0.98 (0.93-1.03)		0.95 (0.91-0.99)		1.09 (1.06-1.12)		0.97 (0.94-1.00)		0.93 (0.91-0.95)	
FLUency user (ref: non-FLUency)^d^			6.00 (5.73-6.29)		0.65 (0.63-0.66)		0.72 (0.70-0.73)		1.31 (1.28-1.34)		0.67 (0.65-0.68)
**Household composition (ref: adult-only)^e^**											
	Child-only	0.48 (0.46-0.49)		0.49 (0.48-0.51)		0.51 (0.50-0.52)		0.66 (0.64-0.67)		0.70 (0.69-0.71)	
	Multigenerational	0.58 (0.57-0.60)		0.53 (0.51-0.54)		0.50 (0.50-0.51)		0.56 (0.55-0.57)		0.67 (0.66-0.68)	
**Region (ref: 1 [Northeast])^f^**											
	2 (DC, MD, WV, DE, NJ, PA, VA)	0.94 (0.90-0.98)		0.88 (0.84-0.91)		0.90 (0.88-0.92)		0.91 (0.89-0.93)		0.85 (0.84-0.87)	
	3 (GA, FL, NC, SC)	1.04 (1.00-1.09)		0.86 (0.83-0.90)		0.83 (0.81-0.85)		0.90 (0.88-0.92)		1.04 (1.02-1.07)	
	4 (KY, TN, AL, MS)	0.92 (0.87-0.98)		0.86 (0.82-0.90)		0.80 (0.77-0.83)		0.87 (0.84-0.90)		0.96 (0.93-0.99)	
	5 (IL, WI, IN, MI, MN, OH)	0.99 (0.95-1.03)		0.87 (0.84-0.91)		0.88 (0.86-0.90)		1.01 (0.99-1.03)		0.92 (0.90-0.94)	
	6 (OK, AR, LA, NM, TX)	0.96 (0.93-1.01)		0.80 (0.77-0.83)		0.82 (0.80-0.84)		0.79 (0.77-0.81)		0.98 (0.96-1.00)	
	7 (NE, IA, KS, MO)	0.88 (0.84-0.93)		0.77 (0.73-0.80)		0.82 (0.79-0.85)		0.93 (0.90-0.96)		0.84 (0.82-0.87)	
	8 (MT, ND, WY, CO, SD, UT)	0.90 (0.84-0.97)		0.91 (0.85-0.96)		0.89 (0.85-0.92)		0.92 (0.89-0.95)		0.87 (0.84-0.89)	
	9 (CA, NV, AZ, HI)	1.03 (0.99-1.07)		0.98 (0.95-1.02)		0.87 (0.85-0.89)		0.81 (0.79-0.82)		0.95 (0.93-0.97)	
	10 (AK, ID, OR, WA)	1.01 (0.94-1.07)		0.79 (0.75-0.84)		0.97 (0.94-1.00)		0.84 (0.81-0.86)		0.98 (0.95-1.01)	

^a^Each study period consisted of a unique population and was analyzed separately. Values shown are the adjusted incidence rate ratios for temperature-taking and their associated 95% CIs. Reference groups are listed next to the name of the predictor.

^b^Percentage of population living below the 100% federal poverty level at the census tract level from the 2015-2019 American Community Survey.

^c^Categorized as urban if the census tract was part of an urbanized area of 50,000 or more people based on the 2010 US Census.

^d^Received the thermometer through Kinsa’s school distribution and engagement program, FLUency.

^e^Based on ages of profiles associated with the device, with child-only households representing devices where a parent has made profiles for their children but not themself.

^f^Classified using the Centers for Disease Control and Prevention National Center For Chronic Disease Prevention and Health Promotion Regions (corresponding state abbreviations are in parentheses).

### Percent of Readings With Fever Regression

During all study periods examined, users aged 2-5 and 6-11 years had higher odds of having a feverish reading compared to users aged 19-30 (Table 3). Users aged 31-60 years and users aged over 60 years had significantly lower odds of having a feverish reading during all study periods. Users aged over 60 years had the lowest odds of having a feverish reading during wave 1 of COVID-19, with 0.26 (95% CI 0.25-0.28) times the odds compared to users aged 19-30 years.

Men had significantly increased odds of having a reading that was feverish compared to women during all periods, and this increased with each subsequent period (Table 3). By wave 3 of COVID-19, men had 27% (95% CI 24%-29%) higher odds of a feverish reading compared to women. Urban users had elevated odds of feverish readings compared to rural users in influenza offseason (OR 1.05, 95% CI 1.01-1.10) and influenza season (OR 1.12, 95% CI 1.09-1.16) (Table 3). This relation shifted during wave 1 of COVID-19 when urban users had 0.90 (95% CI 0.86-0.94) times the odds of feverish readings as rural users. There was no association during wave 2, but by wave 3, urban users again had increased odds of feverish readings compared to rural users (OR 1.18, 95% CI 1.12-1.24).

**Table 3:** Characteristics associated with proportion of feverish readings from multivariable mixed-effects logistic regressions, May 2019-February 2021.

Variables		Odds ratio (95% CI)^a^				
		Offseason 2019 (May 1, 2019, to October 31, 2019)	Flu season (November 1, 2019, to February 2, 2020)	Wave 1 of COVID-19 (February 3, 2020, to May 31, 2020)	Wave 2 of COVID-19 (June 1, 2020, to October 31, 2020)	Wave 3 of COVID-19 (November 1, 2020, to February 28, 2021)
**Age (ref: 19-30 years)**						
	0-1 years	0.56 (0.53-0.58)	0.83 (0.79-0.86)	1.05 (1.00-1.10)	2.03 (1.93-2.14)	2.09 (1.98-2.21)
	2-5 years	1.69 (1.61-1.76)	1.61 (1.55-1.67)	1.93 (1.86-2.00)	2.57 (2.46-2.69)	2.01 (1.93-2.10)
	6-11 years	1.93 (1.85-2.02)	2.08 (2.00-2.15)	1.80 (1.74-1.87)	1.37 (1.30-1.43)	1.09 (1.04-1.14)
	12-18 years	1.33 (1.25-1.41)	1.41 (1.35-1.47)	1.26 (1.20-1.31)	0.95 (0.90-1.01)	0.73 (0.69-0.77)
	31-60 years	0.85 (0.82-0.89)	0.83 (0.80-0.86)	0.73 (0.71-0.76)	0.80 (0.77-0.84)	0.93 (0.90-0.97)
	>60 years	0.73 (0.66-0.81)	0.53 (0.48-0.58)	0.26 (0.25-0.28)	0.48 (0.45-0.52)	0.65 (0.62-0.69)
Gender, men (ref: women)		1.06 (1.04-1.08)	1.06 (1.05-1.08)	1.06 (1.05-1.08)	1.14 (1.12-1.17)	1.27 (1.24-1.29)
Density, urban (ref: rural)^b^		1.05 (1.01-1.10)	1.12 (1.09-1.16)	0.90 (0.86-0.94)	1.00 (0.95-1.06)	1.18 (1.12-1.24)
**Poverty (ref: 0 to <10%)^c^**						
	10% to <20%	1.00 (0.96-1.04)	1.02 (0.99-1.05)	1.14 (1.10-1.19)	1.19 (1.13-1.25)	1.15 (1.10-1.21)
	20% to <30%	1.00 (0.95-1.06)	1.05 (1.01-1.10)	1.18 (1.11-1.25)	1.29 (1.20-1.40)	1.26 (1.17-1.35)
	≥30%	0.99 (0.92-1.08)	1.02 (0.96-1.08)	0.99 (0.91-1.07)	1.30 (1.17-1.43)	1.26 (1.15-1.38)
FLUency user (ref: non-FLUency)^d^		0.19 (0.17-0.21)	0.80 (0.78-0.83)	1.84 (1.74-1.94)	0.72 (0.66-0.78)	0.59 (0.55-0.63)
**Household composition (ref: adult-only)^e^**						
	Child-only	2.21 (2.10-2.33)	1.80 (1.73-1.88)	13.66 (13.04-14.31)	5.83 (5.49-6.20)	3.08 (2.91-3.26)
	Multigenerational	1.60 (1.52-1.69)	1.51 (1.45-1.58)	4.36 (4.16-4.57)	2.62 (2.46-2.79)	1.98 (1.86-2.09)
**Region (ref: 1 [Northeast])^f^**						
	2 (DC, MD, WV, DE, NJ, PA, VA)	1.00 (0.94-1.07)	0.99 (0.94-1.05)	1.17 (1.10-1.26)	1.34 (1.22-1.46)	1.02 (0.94-1.11)
	3 (GA, FL, NC, SC)	1.02 (0.95-1.09)	1.06 (1.00-1.12)	1.25 (1.16-1.34)	1.89 (1.72-2.07)	1.56 (1.43-1.71)
	4 (KY, TN, AL, MS)	0.93 (0.85-1.02)	0.89 (0.83-0.95)	1.23 (1.12-1.36)	2.06 (1.81-2.34)	1.77 (1.57-1.99)
	5 (IL, WI, IN, MI, MN, OH)	0.85 (0.80-0.91)	0.88 (0.83-0.92)	1.20 (1.13-1.28)	1.24 (1.14-1.34)	1.10 (1.02-1.19)
	6 (OK, AR, LA, NM, TX)	0.97 (0.91-1.04)	1.00 (0.95-1.05)	1.27 (1.19-1.37)	1.98 (1.81-2.17)	1.73 (1.59-1.89)
	7 (NE, IA, KS, MO)	0.81 (0.74-0.88)	0.87 (0.82-0.93)	1.24 (1.13-1.35)	1.31 (1.17-1.48)	1.37 (1.22-1.54)
	8 (MT, ND, WY, CO, SD, UT)	0.81 (0.73-0.90)	0.85 (0.78-0.93)	0.81 (0.72-0.90)	1.12 (0.98-1.28)	1.10 (0.97-1.25)
	9 (CA, NV, AZ, HI)	0.99 (0.93-1.05)	1.07 (1.02-1.13)	0.99 (0.93-1.06)	1.57 (1.45-1.70)	1.42 (1.31-1.53)
	10 (AK, ID, OR, WA)	0.79 (0.72-0.88)	0.91 (0.84-1.00)	0.58 (0.53-0.64)	0.73 (0.64-0.82)	0.86 (0.76-0.98)

^a^Each study period consisted of a unique population and was analyzed separately. Values shown are the odds ratios with their associated 95% CIs. Reference groups are listed next to the name of the predictor.

^b^Percentage of population living below the 100% federal poverty level at the census tract level from the 2015-2019 American Community Survey.

^c^Categorized as urban if the census tract was part of an urbanized area of 50,000 or more people based on the 2010 US Census.

^d^Received the thermometer through Kinsa’s school distribution and engagement program, FLUency.

^e^Based on ages of profiles associated with the device, with child-only households representing devices where a parent has made profiles for their children but not themself.

^f^Classified using the Centers for Disease Control and Prevention National Center For Chronic Disease Prevention and Health Promotion Regions.

## Discussion

### Principal Findings

Using data collected from smart thermometers, we analyzed temperature-taking behaviors through periods prior to and during the COVID-19 pandemic. We found that both the frequency of readings and proportion of feverish readings varied with age group, gender, urban/rural status, and circulating illness. The differences observed between demographic groups reflect a combination of changes in both actual illness risk and perceived risk that can only fully be understood through dual examination of the number of readings and the percent of those readings with a fever.

Users aged over 60 years experienced the largest shift in temperature-taking behaviors over the study period: during the influenza season, they were less likely to take their temperatures than young adults (aged 19-30 years), whereas through all three waves of COVID-19 assessed in the study, they had an elevated frequency of temperature-taking and reduced proportion of feverish readings.

### Comparison With Prior Work

The significant switch in behavior among older adult users could reflect increases in perceived risk during COVID-19. Adults 65 years and older have higher odds of COVID-19–related concerns [2], and outcomes of hospitalization and death have been the most severe among older adults [27]. Additionally, individuals are more likely to take preventative action if they have a higher perceived risk of a negative health outcome [28]. We hypothesize that the observed shift in temperature-taking behaviors among older adults is related to increased monitoring for signs of possible COVID-19 infection, given its potential severe outcomes, even in the absence of other symptoms. The proportion of older adults among Kinsa users also increased over the three waves of COVID-19 (Table 1), likely as older adults bought and used thermometers more during the pandemic due to increases in perceived risk.

Users aged 2-5 years and 6-11 years had both increased rates of temperature-taking frequency and increased odds of those readings being feverish compared to young adults during all study periods. Child temperature-taking likely reflects parental behavior and concern. Typically, we would expect that as temperature-taking frequency increases, the percent of feverish readings would decrease because the denominator becomes larger. However, our findings suggest that during the COVID-19 pandemic, children were more likely than young adults to have a fever of any origin, as children’s temperatures were taken more often and each of those readings had a higher odds of being feverish. Similar to testing for COVID-19, only by examining both the frequency of readings and the percent of those readings with a fever can patterns of behavior and disease be separated [29]. Before the COVID-19 pandemic, children were found to experience febrile illness more often in an average year than adults [16], which may contribute to the observed increased odds of feverish readings. Because children were less likely to experience severe disease from COVID-19 [30], it is possible that child temperature-taking was undermeasured due to reduced perceived risk from caretakers. A reduced risk of severe outcomes in children may also explain why children were tested for COVID-19 less often than adults [18].

In line with previous research on most personal health behaviors [31,32], men took their temperature less frequently than women across all study periods (Table 2). Lower rates of monitoring likely explain why when men did take their temperature, they had higher odds of being feverish (Table 3). Prior research has found that men were less likely to pay attention to global pandemics than women [33] and less likely to be concerned about COVID-19 during the first two waves of the pandemic [2]. Globally, women have been tested for COVID-19 more frequently than men [34]. Similarly, within the United States, men were tested for COVID-19 less often and had a higher test positivity rate than women [35]. The increased odds of fever we observed in men likely reflects both a decreased perceived risk among men and increased temperature-taking behavior among women.

Urban users had higher rates of temperature-taking than rural users during all study periods, except for wave 2 of the pandemic. Additionally, during wave 1 of COVID-19, researchers found that urban residents reported increases in other health behaviors such as mask wearing and social distancing compared to rural residents [36]. It is likely that either urban users overmonitored their temperatures or rural users undermonitored theirs, since the odds of urban users having feverish readings decreased during wave 1 compared to rural users. Rural areas were more heavily impacted by wave 2 of COVID-19 in terms of cases, hospitalizations, and deaths [37]; accordingly, urban users may have had a decreased perceived risk relative to rural users, leading to their decrease in temperature-taking frequency during this time.

Our study has many strengths that contribute to the literature on surveillance and health behavior. We have a large sample size across multiple illness seasons. Because our behavioral data are not reliant on self-report, we gained an accurate, real-time measure of temperature-taking behavior that is not subject to recall bias. Furthermore, fever data from smart thermometers have been correlated with both COVID-19 cases and influenza-like illness levels [16-19]. Unlike cross-sectional studies that were initiated after the start of the COVID-19 pandemic, we examined data across a previous influenza season, offseason, and multiple waves of COVID-19.

### Limitations

There are also limitations to our study design worth noting. The series of cross-sectional studies do not represent the same population over time and therefore could reflect changes in the user populations rather than changes in user behavior. Future studies should follow a cohort across multiple illness seasons. Because we condition on owning and using a smart thermometer, our study population may not be representative of the US population that does not own a smartphone. However, we capture a wide range of socioeconomic status (Table 1). We also did not have individual-level data on poverty, race, education, or occupation, which could confound some of the observed associations. Additional factors, besides demographics and illness risk, may have impacted the results if a user took a temperature each time they had a possible fever. Users also could have misassigned fevers to family members if they forgot to switch profiles before taking a temperature.

### Conclusions

Unlike survey data, temperature-taking provides real-time insights into individual behaviors and concerns about circulating infectious disease. Thermometer usage rises with disease circulation, as the highest frequencies were observed during influenza season and wave 1 of COVID-19. Demographic groups react differently to changes in disease levels, with rural residents and young men taking their temperature less often. These behavioral shifts likely reflect perceived risk more than actual risk. Future studies should investigate how upstream factors such as media coverage impact perceived risk and temperature-taking behavior. Public health surveillance should consider how these behaviors affect testing and health monitoring in interpreting disease levels in different demographic groups.

## Appendix

Multimedia Appendix 1 Sensitivity analysis examining demographic characteristics associated with temperature-taking frequency measured by the number of days with a reading. Results of the mixed-effects negative binomial regressions for each period. Values shown are the adjusted incidence rate ratios and their associated 95% CIs. Reference groups are listed next to the name of the predictor.

## Abbreviations

HAPA: Health Action Process Approach
IRR: incidence rate ratio
OR: odds ratio
