# Supplementary material for: Circulating Illness and Changes in Thermometer Use Behavior: Series of Cross-sectional Analyses
Source: JMIR Form Res. 2022 Sep 8;6(9):e37509. doi: 10.2196/37509 (PMC9506504; doi:10.2196/37509)
Supplement: Multimedia Appendix 1 [file formative_v6i9e37509_app1.docx]

**Multimedia Appendix 1:** The sensitivity analysis examining demographic characteristics associated with temperature-taking frequency measured by the number of days with a reading. Results of the mixed effects negative binomial regressions for each period. Values shown are the adjusted incidence rate ratios and their associated 95% confidence intervals. Reference groups are listed next to the name of the predictor.

|  | Incidence Rate Ratio (95% CI) | |  |  |  |
| --- | --- | --- | --- | --- | --- |
|  | **Offseason 2019** | **Flu Season** | **Wave 1 COVID** | **Wave 2 COVID** | **Wave 3 COVID** |
|  | **(May 1, 2019 –**  **Oct 31, 2019)** | **(Nov 1, 2019 –Feb 2, 2020)** | **(Feb 3, 2020 –**  **May 31, 2020)** | **(Jun 1, 2020 –**  **Oct 31, 2020)** | **(Nov 1, 2020 –**  **Feb 28, 2021)** |
| **Age (ref: 19-30)** |  |  |  |  |  |
| 0-1 years | 2.10 (2.05, 2.16) | 1.46 (1.43, 1.50) | 1.70 (1.67, 1.74) | 1.14 (1.11, 1.16) | 1.25 (1.22, 1.27) |
| 2-5 years | 1.61 (1.57, 1.65) | 1.59 (1.56, 1.63) | 1.39 (1.37, 1.42) | 0.99 (0.97, 1.01) | 1.13 (1.11, 1.15) |
| 6-11 years | 1.44 (1.41, 1.48) | 1.64 (1.61, 1.67) | 1.29 (1.27, 1.31) | 1.11 (1.09, 1.13) | 1.16 (1.14, 1.18) |
| 12-18 years | 1.20 (1.16, 1.24) | 1.36 (1.33, 1.39) | 1.07 (1.05, 1.09) | 0.96 (0.94, 0.98) | 1.00 (0.98, 1.02) |
| 31-60 years | 0.96 (0.94, 0.99) | 1.02 (1.00, 1.04) | 1.38 (1.36, 1.40) | 1.04 (1.03, 1.06) | 1.14 (1.12, 1.15) |
| >60 years | 1.28 (1.22, 1.34) | 1.21 (1.16, 1.26) | 2.35 (2.32, 2.39) | 1.51 (1.49, 1.54) | 1.62 (1.60, 1.65) |
| **Gender (ref: women)** |  |  |  |  |  |
| Men | 0.93 (0.92, 0.94) | 0.95 (0.94, 0.96) | 0.96 (0.96, 0.97) | 0.99 (0.98, 1.00) | 0.98 (0.97, 0.98) |
| **Density (ref: rural)^a^** |  |  |  |  |  |
| Urban | 1.07 (1.05, 1.09) | 1.08 (1.06, 1.09) | 1.06 (1.05, 1.07) | 0.92 (0.90, 0.93) | 1.00 (0.99, 1.01) |
| **Poverty (ref: 0-<10%)^b^** |  |  |  |  |  |
| 10-<20% | 0.97 (0.95, 0.98) | 0.97 (0.96, 0.99) | 1.01 (1.00, 1.02) | 1.00 (0.98, 1.01) | 1.00 (0.99, 1.01) |
| 20-<30% | 0.95 (0.93, 0.98) | 0.97 (0.95, 0.99) | 1.05 (1.03, 1.07) | 0.97 (0.95, 0.99) | 0.95 (0.94, 0.97) |
| **≥**30% | 0.97 (0.93, 1.00) | 0.96 (0.93, 0.99) | 1.10 (1.07, 1.13) | 0.97 (0.95, 0.99) | 0.96 (0.94, 0.98) |
| **FLUency (ref: non-FLUency)^c^** |  |  |  |  |  |
| FLUency user | 10.46 (10.10, 10.84) | 0.89 (0.88, 0.91) | 0.73 (0.72, 0.75) | 1.56 (1.53, 1.60) | 0.84 (0.83, 0.85) |
| **Household Composition (ref: adult-only)^d^** |  |  |  |  |  |
| Child-only | 0.52 (0.51, 0.53) | 0.58 (0.57, 0.59) | 0.42 (0.41, 0.43) | 0.63 (0.62, 0.64) | 0.71 (0.70, 0.72) |
| Multi-generational | 0.70 (0.69, 0.72) | 0.72 (0.71, 0.74) | 0.58 (0.58, 0.59) | 0.66 (0.64, 0.67) | 0.72 (0.71, 0.73) |
| **Region (ref: 1 [Northeast])^e^** |  |  |  |  |  |
| 2 (DC, MD, WV, DE, NJ, PA, VA) | 0.96 (0.93, 0.99) | 0.89 (0.87, 0.92) | 0.90 (0.88, 0.91) | 0.82 (0.80, 0.84) | 0.86 (0.84, 0.87) |
| 3 (GA, FL, NC, SC) | 1.00 (0.97, 1.03) | 0.85 (0.83, 0.87) | 0.83 (0.81, 0.84) | 0.76 (0.74, 0.77) | 0.95 (0.93, 0.97) |
| 4 (KY, TN, AL, MS) | 0.93 (0.89, 0.97) | 0.86 (0.84, 0.89) | 0.79 (0.77, 0.81) | 0.72 (0.70, 0.74) | 0.86 (0.84, 0.89) |
| 5 (IL, WI, IN, MI, MN, OH) | 0.97 (0.94, 1.00) | 0.87 (0.85, 0.89) | 0.86 (0.85, 0.88) | 0.92 (0.90, 0.93) | 0.89 (0.87, 0.91) |
| 6 (OK, AR, LA, NM, TX) | 0.94 (0.91, 0.96) | 0.80 (0.78, 0.82) | 0.79 (0.77, 0.80) | 0.66 (0.64, 0.67) | 0.88 (0.86, 0.90) |
| 7 (NE, IA, KS, MO) | 0.86 (0.82, 0.89) | 0.79 (0.76, 0.81) | 0.80 (0.78, 0.82) | 0.79 (0.77, 0.81) | 0.78 (0.76, 0.80) |
| 8 (MT, ND, WY, CO, SD, UT) | 0.89 (0.85, 0.94) | 0.89 (0.86, 0.93) | 0.91 (0.89, 0.94) | 0.85 (0.82, 0.87) | 0.84 (0.82, 0.87) |
| 9 (CA, NV, AZ, HI) | 0.96 (0.93, 0.99) | 0.92 (0.90, 0.94) | 0.89 (0.87, 0.90) | 0.73 (0.72, 0.74) | 0.90 (0.88, 0.92) |
| 10 (AK, ID, OR, WA) | 1.03 (0.98, 1.08) | 0.81 (0.78, 0.85) | 1.06 (1.03, 1.09) | 0.83 (0.80, 0.85) | 1.00 (0.97, 1.03) |

^a^ Percentage of population living below 100% federal poverty level at Census tract level, from the 2015–2019 American Community Survey.

^b^Categorized as urban if census tract was part of an urbanized area of 50,000 or more people based on 2010 US Census

^c^Received thermometer through Kinsa’s school distribution and engagement program, FLUency

^d^Based on ages of profiles associated with the device with child-only households representing devices where a parent has made profiles for their children but not themself

^e^Classified using CDC National Center For Chronic Disease Prevention and Health Promotion Regions
